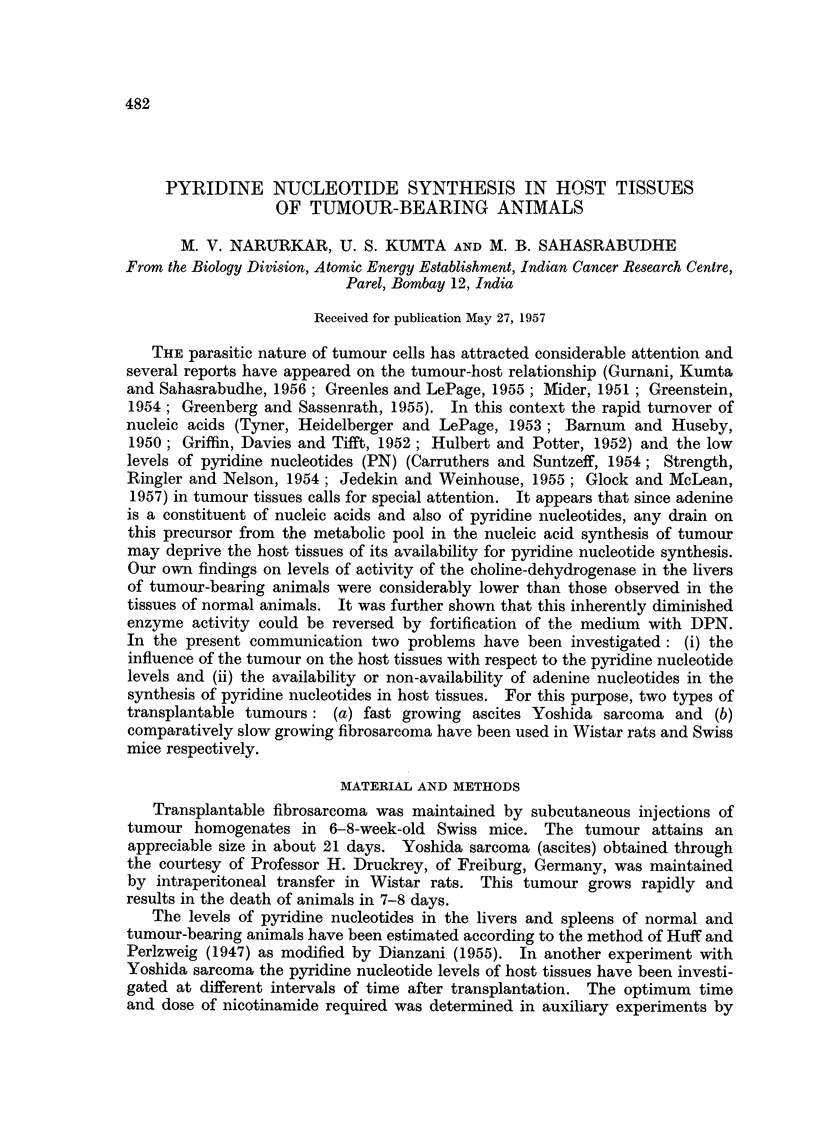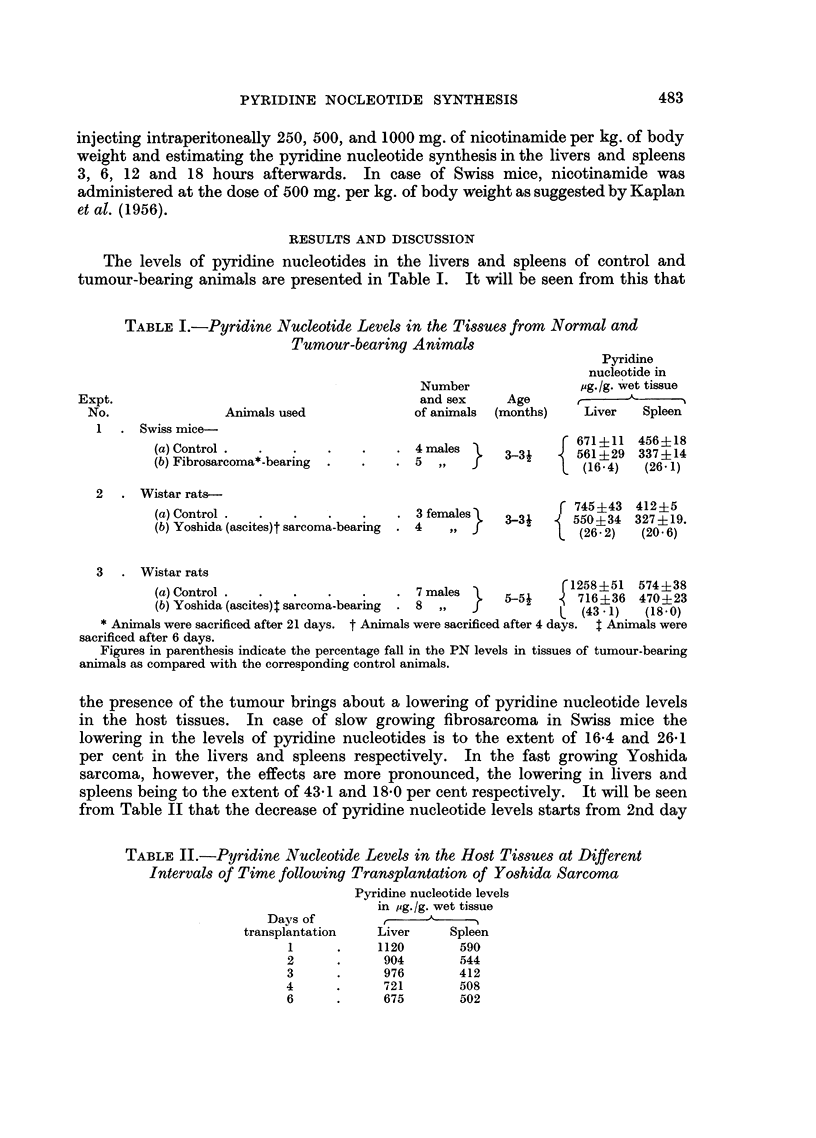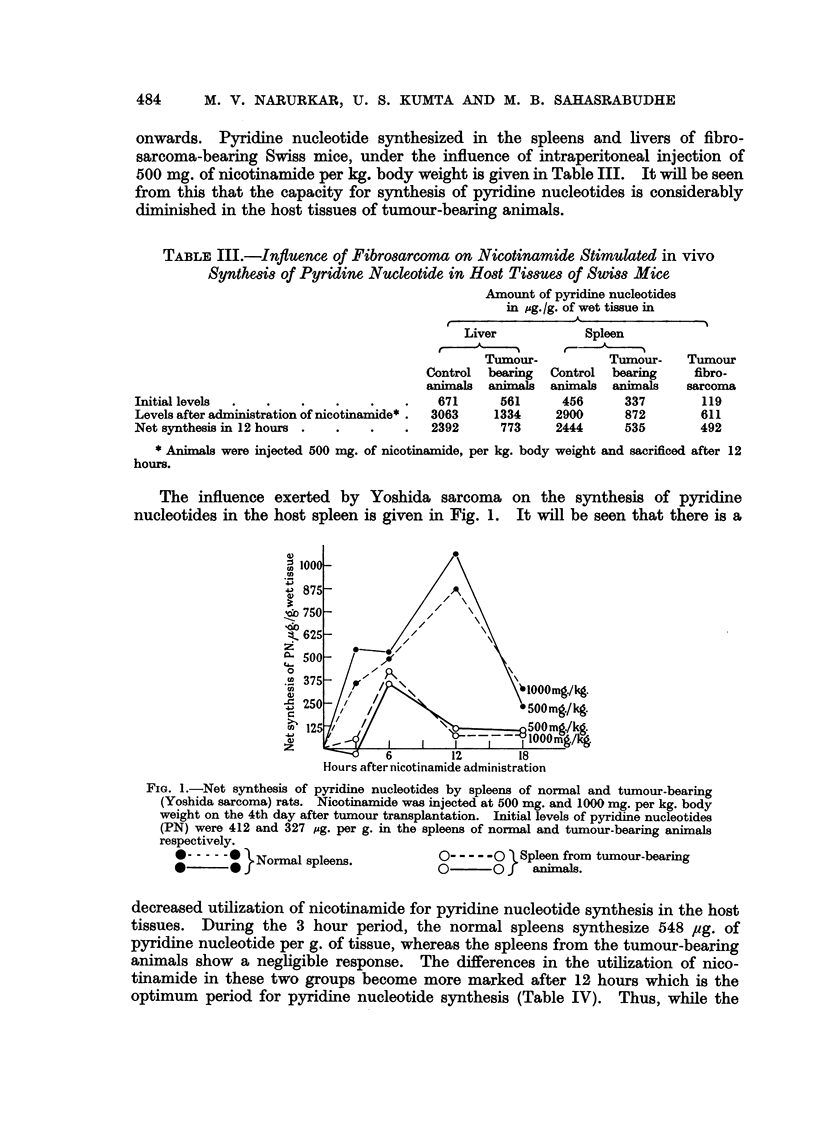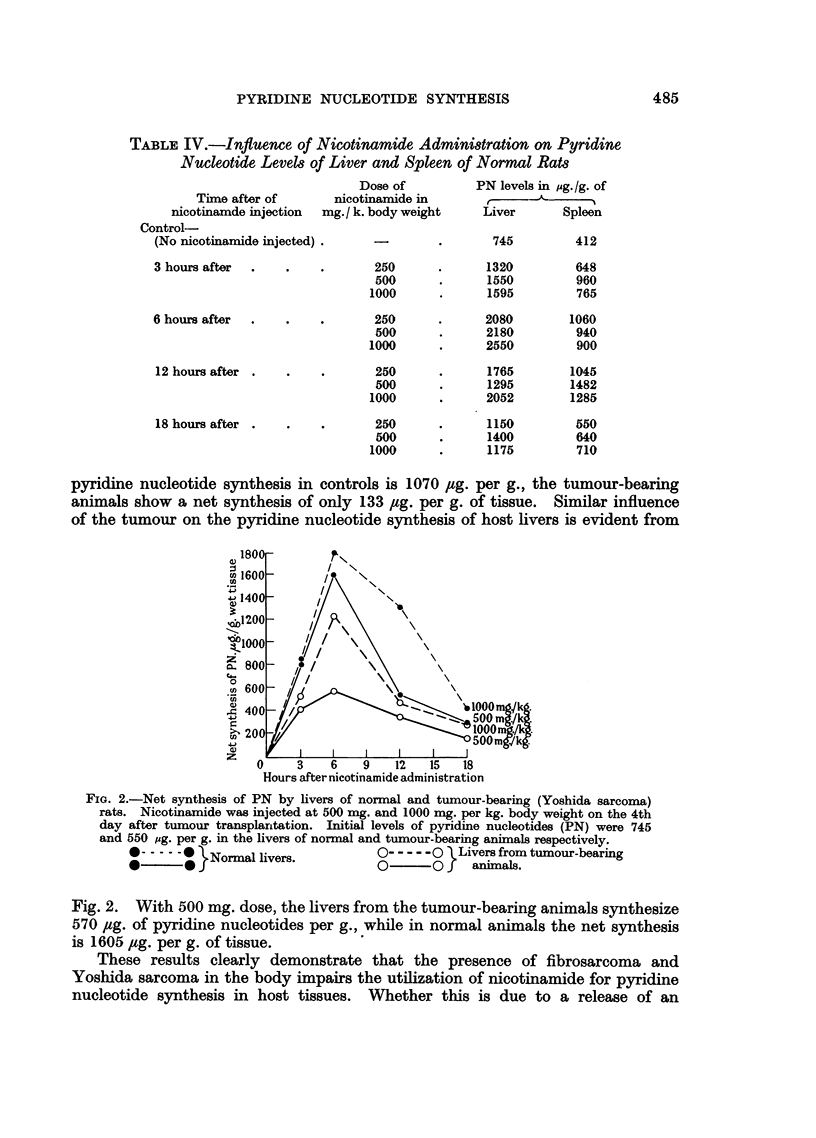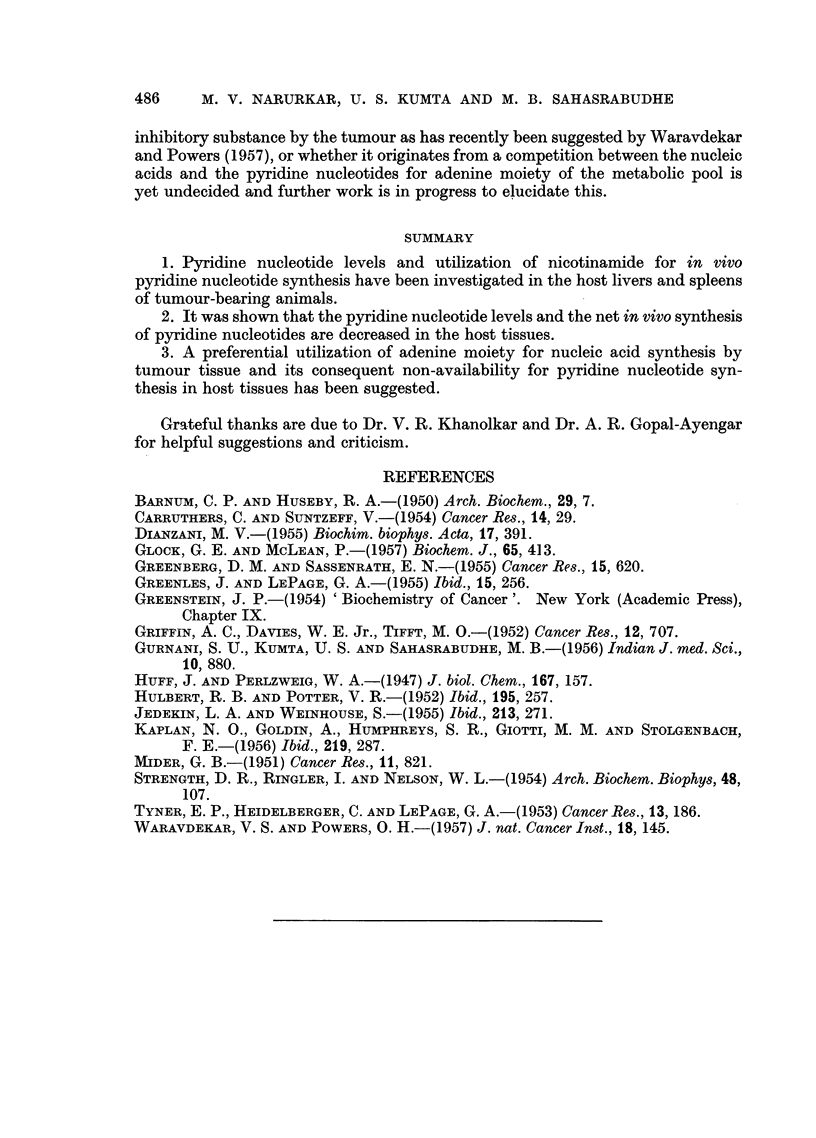# Pyridine Nucleotide Synthesis in Host Tissues of Tumour-Bearing Animals

**DOI:** 10.1038/bjc.1957.58

**Published:** 1957-09

**Authors:** M. V. Narurkar, U. S. Kumta, M. B. Sahasrabudhe


### PYRIDINE NUCLEOTIDE SYNTHESIS IN HOST TISSUES OF TUMOUR-BEARING ANIMALS

M. V. NARURKAR, U. S. KUMTA AND M. B. SAHASRABUDHE

*From the Biology Division, Atomic Energy Establishment, Indian Cancer Research Centre, Parel, Bombay 12, India*

Received for publication May 27, 1957

THE parasitic nature of tumour cells has attracted considerable attention and several reports have appeared on the tumour-host relationship (Gurnani, Kumta and Sahasrabudhe, 1956 ; Greenles and LePage, 1955 ; Mider, 1951 ; Greenstein, 1954 ; Greenberg and Sassenrath, 1955). In this context the rapid turnover of nucleic acids (Tyner, Heidelberger and LePage, 1953 ; Barnum and Huseby, 1950 ; Griffin, Davies and Tifft, 1952 ; Hulbert and Potter, 1952) and the low levels of pyridine nucleotides (PN) (Carruthers and Suntzeff, 1954 ; Strength, Ringler and Nelson, 1954 ; Jedekin and Weinhouse, 1955 ; Glock and McLean, 1957) in tumour tissues calls for special attention. It appears that since adenine is a constituent of nucleic acids and also of pyridine nucleotides, any drain on this precursor from the metabolic pool in the nucleic acid synthesis of tumour may deprive the host tissues of its availability for pyridine nucleotide synthesis. Our own findings on levels of activity of the choline-dehydrogenase in the livers of tumour-bearing animals were considerably lower than those observed in the tissues of normal animals. It was further shown that this inherently diminished enzyme activity could be reversed by fortification of the medium with DPN. In the present communication two problems have been investigated : (i) the influence of the tumour on the host tissues with respect to the pyridine nucleotide levels and (ii) the availability or non-availability of adenine nucleotides in the synthesis of pyridine nucleotides in host tissues. For this purpose, two types of transplantable tumours : (*a*) fast growing ascites Yoshida sarcoma and (*b*) comparatively slow growing fibrosarcoma have been used in Wistar rats and Swiss mice respectively.

### MATERIAL AND METHODS

Transplantable fibrosarcoma was maintained by subcutaneous injections of tumour homogenates in 6–8-week-old Swiss mice. The tumour attains an appreciable size in about 21 days. Yoshida sarcoma (ascites) obtained through the courtesy of Professor H. Druckrey, of Freiburg, Germany, was maintained by intraperitoneal transfer in Wistar rats. This tumour grows rapidly and results in the death of animals in 7–8 days.

The levels of pyridine nucleotides in the livers and spleens of normal and tumour-bearing animals have been estimated according to the method of Huff and Perlzweig (1947) as modified by Dianzani (1955). In another experiment with Yoshida sarcoma the pyridine nucleotide levels of host tissues have been investigated at different intervals of time after transplantation. The optimum time and dose of nicotinamide required was determined in auxiliary experiments by injecting intraperitoneally 250, 500, and 1000 mg. of nicotinamide per kg. of body weight and estimating the pyridine nucleotide synthesis in the livers and spleens 3, 6, 12 and 18 hours afterwards. In case of Swiss mice, nicotinamide was administered at the dose of 500 mg. per kg. of body weight as suggested by Kaplan *et al.* (1956).

### RESULTS AND DISCUSSION

The levels of pyridine nucleotides in the livers and spleens of control and tumour-bearing animals are presented in Table I. It will be seen from this that

TABLE I.—*Pyridine Nucleotide Levels in the Tissues from Normal and Tumour-bearing Animals*

| Expt. No. | Animals used | Number and sex of animals | Age (months) | Pyridine nucleotide in μg./g. wet tissue: Liver | Spleen |
|---|---|---|---|---|---|
| 1 | Swiss mice— | | | | |
| | (a) Control | 4 males | 3–3½ | 671±11 | 456±18 |
| | (b) Fibrosarcoma*-bearing | 5 „ | | 561±29 | 337±14 |
| | | | | (16·4) | (26·1) |
| 2 | Wistar rats— | | | | |
| | (a) Control | 3 females | 3–3½ | 745±43 | 412±5 |
| | (b) Yoshida (ascites)† sarcoma-bearing | 4 „ | | 550±34 | 327±19. |
| | | | | (26·2) | (20·6) |
| 3 | Wistar rats | | | | |
| | (a) Control | 7 males | 5–5½ | 1258±51 | 574±38 |
| | (b) Yoshida (ascites)‡ sarcoma-bearing | 8 „ | | 716±36 | 470±23 |
| | | | | (43·1) | (18·0) |

* Animals were sacrificed after 21 days. † Animals were sacrificed after 4 days. ‡ Animals were sacrificed after 6 days.

Figures in parenthesis indicate the percentage fall in the PN levels in tissues of tumour-bearing animals as compared with the corresponding control animals.

the presence of the tumour brings about a lowering of pyridine nucleotide levels in the host tissues. In case of slow growing fibrosarcoma in Swiss mice the lowering in the levels of pyridine nucleotides is to the extent of 16·4 and 26·1 per cent in the livers and spleens respectively. In the fast growing Yoshida sarcoma, however, the effects are more pronounced, the lowering in livers and spleens being to the extent of 43·1 and 18·0 per cent respectively. It will be seen from Table II that the decrease of pyridine nucleotide levels starts from 2nd day

TABLE II.—*Pyridine Nucleotide Levels in the Host Tissues at Different Intervals of Time following Transplantation of Yoshida Sarcoma*

| Days of transplantation | Pyridine nucleotide levels in μg./g. wet tissue: Liver | Spleen |
|---|---|---|
| 1 | 1120 | 590 |
| 2 | 904 | 544 |
| 3 | 976 | 412 |
| 4 | 721 | 508 |
| 6 | 675 | 502 |

onwards. Pyridine nucleotide synthesized in the spleens and livers of fibrosarcoma-bearing Swiss mice, under the influence of intraperitoneal injection of 500 mg. of nicotinamide per kg. body weight is given in Table III. It will be seen from this that the capacity for synthesis of pyridine nucleotides is considerably diminished in the host tissues of tumour-bearing animals.

TABLE III.—*Influence of Fibrosarcoma on Nicotinamide Stimulated* in vivo *Synthesis of Pyridine Nucleotide in Host Tissues of Swiss Mice*

| | Amount of pyridine nucleotides in μg./g. of wet tissue in | | | | |
|---|---|---|---|---|---|
| | Liver | | Spleen | | |
| | Control animals | Tumour-bearing animals | Control animals | Tumour-bearing animals | Tumour fibro-sarcoma |
| Initial levels . . . . . . . | 671 | 561 | 456 | 337 | 119 |
| Levels after administration of nicotinamide* . | 3063 | 1334 | 2900 | 872 | 611 |
| Net synthesis in 12 hours . . . . | 2392 | 773 | 2444 | 535 | 492 |

* Animals were injected 500 mg. of nicotinamide, per kg. body weight and sacrificed after 12 hours.

The influence exerted by Yoshida sarcoma on the synthesis of pyridine nucleotides in the host spleen is given in Fig. 1. It will be seen that there is a

FIG. 1.—Net synthesis of pyridine nucleotides by spleens of normal and tumour-bearing (Yoshida sarcoma) rats. Nicotinamide was injected at 500 mg. and 1000 mg. per kg. body weight on the 4th day after tumour transplantation. Initial levels of pyridine nucleotides (PN) were 412 and 327 μg. per g. in the spleens of normal and tumour-bearing animals respectively.

●- - - - -● }
●———● } Normal spleens.

○- - - - -○ }
○———○ } Spleen from tumour-bearing animals.

decreased utilization of nicotinamide for pyridine nucleotide synthesis in the host tissues. During the 3 hour period, the normal spleens synthesize 548 μg. of pyridine nucleotide per g. of tissue, whereas the spleens from the tumour-bearing animals show a negligible response. The differences in the utilization of nicotinamide in these two groups become more marked after 12 hours which is the optimum period for pyridine nucleotide synthesis (Table IV). Thus, while the

TABLE IV.—*Influence of Nicotinamide Administration on Pyridine Nucleotide Levels of Liver and Spleen of Normal Rats*

| Time after of nicotinamde injection | Dose of nicotinamide in mg./k. body weight | PN levels in μg./g. of Liver | Spleen |
|---|---|---|---|
| Control— (No nicotinamide injected) | — | 745 | 412 |
| 3 hours after | 250 | 1320 | 648 |
| | 500 | 1550 | 960 |
| | 1000 | 1595 | 765 |
| 6 hours after | 250 | 2080 | 1060 |
| | 500 | 2180 | 940 |
| | 1000 | 2550 | 900 |
| 12 hours after | 250 | 1765 | 1045 |
| | 500 | 1295 | 1482 |
| | 1000 | 2052 | 1285 |
| 18 hours after | 250 | 1150 | 550 |
| | 500 | 1400 | 640 |
| | 1000 | 1175 | 710 |

pyridine nucleotide synthesis in controls is 1070 μg. per g., the tumour-bearing animals show a net synthesis of only 133 μg. per g. of tissue. Similar influence of the tumour on the pyridine nucleotide synthesis of host livers is evident from

FIG. 2.—Net synthesis of PN by livers of normal and tumour-bearing (Yoshida sarcoma) rats. Nicotinamide was injected at 500 mg. and 1000 mg. per kg. body weight on the 4th day after tumour transplantation. Initial levels of pyridine nucleotides (PN) were 745 and 550 μg. per g. in the livers of normal and tumour-bearing animals respectively.
●- - - - -● / ●———● } Normal livers. ○- - - - -○ / ○———○ } Livers from tumour-bearing animals.

Fig. 2. With 500 mg. dose, the livers from the tumour-bearing animals synthesize 570 μg. of pyridine nucleotides per g., while in normal animals the net synthesis is 1605 μg. per g. of tissue.

These results clearly demonstrate that the presence of fibrosarcoma and Yoshida sarcoma in the body impairs the utilization of nicotinamide for pyridine nucleotide synthesis in host tissues. Whether this is due to a release of an inhibitory substance by the tumour as has recently been suggested by Waravdekar and Powers (1957), or whether it originates from a competition between the nucleic acids and the pyridine nucleotides for adenine moiety of the metabolic pool is yet undecided and further work is in progress to elucidate this.

### SUMMARY

1. Pyridine nucleotide levels and utilization of nicotinamide for *in vivo* pyridine nucleotide synthesis have been investigated in the host livers and spleens of tumour-bearing animals.

2. It was shown that the pyridine nucleotide levels and the net *in vivo* synthesis of pyridine nucleotides are decreased in the host tissues.

3. A preferential utilization of adenine moiety for nucleic acid synthesis by tumour tissue and its consequent non-availability for pyridine nucleotide synthesis in host tissues has been suggested.

Grateful thanks are due to Dr. V. R. Khanolkar and Dr. A. R. Gopal-Ayengar for helpful suggestions and criticism.

### REFERENCES

Barnum, C. P. and Huseby, R. A.—(1950) *Arch. Biochem.*, **29**, 7.

Carruthers, C. and Suntzeff, V.—(1954) *Cancer Res.*, **14**, 29.

Dianzani, M. V.—(1955) *Biochim. biophys. Acta*, **17**, 391.

Glock, G. E. and McLean, P.—(1957) *Biochem. J.*, **65**, 413.

Greenberg, D. M. and Sassenrath, E. N.—(1955) *Cancer Res.*, **15**, 620.

Greenles, J. and LePage, G. A.—(1955) *Ibid.*, **15**, 256.

Greenstein, J. P.—(1954) ‘Biochemistry of Cancer’. New York (Academic Press), Chapter IX.

Griffin, A. C., Davies, W. E. Jr., Tifft, M. O.—(1952) *Cancer Res.*, **12**, 707.

Gurnani, S. U., Kumta, U. S. and Sahasrabudhe, M. B.—(1956) *Indian J. med. Sci.*, **10**, 880.

Huff, J. and Perlzweig, W. A.—(1947) *J. biol. Chem.*, **167**, 157.

Hulbert, R. B. and Potter, V. R.—(1952) *Ibid.*, **195**, 257.

Jedekin, L. A. and Weinhouse, S.—(1955) *Ibid.*, **213**, 271.

Kaplan, N. O., Goldin, A., Humphreys, S. R., Giotti, M. M. and Stolgenbach, F. E.—(1956) *Ibid.*, **219**, 287.

Mider, G. B.—(1951) *Cancer Res.*, **11**, 821.

Strength, D. R., Ringler, I. and Nelson, W. L.—(1954) *Arch. Biochem. Biophys*, **48**, 107.

Tyner, E. P., Heidelberger, C. and LePage, G. A.—(1953) *Cancer Res.*, **13**, 186.

Waravdekar, V. S. and Powers, O. H.—(1957) *J. nat. Cancer Inst.*, **18**, 145.